# Fermented Foods as a Dietary Source of Live Organisms

**DOI:** 10.3389/fmicb.2018.01785

**Published:** 2018-08-24

**Authors:** Shannon Rezac, Car Reen Kok, Melanie Heermann, Robert Hutkins

**Affiliations:** Department of Food Science and Technology, University of Nebraska—Lincoln, Lincoln, NE, United States

**Keywords:** fermented foods, live microbes, lactic acid bacteria, health benefits, probiotics

## Abstract

The popularity of fermented foods and beverages is due to their enhanced shelf-life, safety, functionality, sensory, and nutritional properties. The latter includes the presence of bioactive molecules, vitamins, and other constituents with increased availability due to the process of fermentation. Many fermented foods also contain live microorganisms that may improve gastrointestinal health and provide other health benefits, including lowering the risk of type two diabetes and cardiovascular diseases. The number of organisms in fermented foods can vary significantly, depending on how products were manufactured and processed, as well as conditions and duration of storage. In this review, we surveyed published studies in which lactic acid and other relevant bacteria were enumerated from the most commonly consumed fermented foods, including cultured dairy products, cheese, fermented sausage, fermented vegetables, soy-fermented foods, and fermented cereal products. Most of the reported data were based on retail food samples, rather than experimentally produced products made on a laboratory scale. Results indicated that many of these fermented foods contained 10^5−7^ lactic acid bacteria per mL or gram, although there was considerable variation based on geographical region and sampling time. In general, cultured dairy products consistently contained higher levels, up to 10^9^/mL or g. Although few specific recommendations and claim legislations for what constitutes a relevant dose exist, the findings from this survey revealed that many fermented foods are a good source of live lactic acid bacteria, including species that reportedly provide human health benefits.

## Introduction

Fermentation has long been used to preserve and enhance the shelf-life, flavor, texture, and functional properties of food (Hutkins, 2018). More recently, the consumption of fermented foods containing live microorganisms has emerged as an important dietary strategy for improving human health (Marco et al., 2017). In general, lactic acid bacteria (LAB) from several genera, including *Lactobacillus, Streptococcus*, and *Leuconostoc* are predominant in fermented foods, but other bacteria as well as yeast and fungi also contribute to food fermentations. Commercially-produced fermented foods also frequently serve as carriers for probiotic bacteria. Despite this interest and the potential public health benefits of these foods, there is still considerable confusion about which fermented foods actually contain live microorganisms, as well as understanding the role of these microbes on the gut microbiome (Slashinski et al., 2012).

Nonetheless, yogurt and other cultured dairy products are generally perceived by consumers as good sources of live and health-promoting organisms (Panahi et al., 2016). Moreover, in a survey of 335 adults, yogurt was the main food associated with probiotic bacteria (Stanczak and Heuberger, 2009). However, the actual concept of fermentation is evidently not so familiar—a survey of 233 college students attending Brescia University College in London, Ontario revealed that nearly two-thirds were unfamiliar with the term “fermented dairy products,” and about the same percent were unsure that several cultured dairy products were fermented (Hekmat and Koba, 2006).

That a particular food or beverage is produced by fermentation does not necessarily indicate that it contains live microorganisms. Bread, beer, wine, and distilled alcoholic beverages require yeasts for fermentation, but the production organisms are either inactivated by heat (in the case of bread and some beers) or are physically removed by filtration or other means (in the case of wine and beer). Moreover, many fermented foods are heat-treated after fermentation to enhance food safety or to extend shelf-life. Thus, fermented sausages are often cooked after fermentation, and soy sauce and sauerkraut and other fermented vegetables are made shelf-stable by thermal processing. Some products, such as many of the commercial pickles and olives, are not fermented at all, but rather are placed into brines containing salt and organic acids. Even non-thermally processed fermented foods may yet contain low levels of live or viable organisms simply due to inhospitable environmental conditions that reduce microbial populations over time. It is important to note, however, that the absence of live microbes in the final product does not preclude a positive functional role. For example, food fermentation microbes may produce vitamins or other bioactive molecules *in situ* or inactivate anti-nutritional factors and yet be absent at the time of consumption.

## Labeling live microbes in fermented foods and beverages

Yogurt, kefir, and other cultured dairy product manufacturers have long promoted the presence of live cultures. Indeed, the “live and active” seal was created by the National Yogurt Association (NYA), for yogurt products in the United States containing at least 100 million cells or cfu per gram at the time of manufacture (Frye and Kilara, 2016). According to the NYA, the “live and active” seal refers only to yogurt cultures, and specifically to the two species that comprise such cultures, *Streptococcus thermophilus* and *Lactobacillus delbrueckii* subsp. *bulgaricus*. However, frozen yogurt, kefir and other cultured dairy products also claim the presence of live and active cultures, even though the microorganisms may be different than those found in yogurt. In the U.S., there is no regulatory requirement to state microbial levels, thus these label declarations are strictly voluntary.

In contrast, in other regions, the number of live microbes present in yogurt and other cultured dairy products must satisfy regulatory requirements. For example, according to the CODEX standards for fermented milk products, the minimum number of starter culture bacteria in yogurt is 10^7^ cfu per g (CODEX STAN 243-2003). If other organisms are indicated on the label, they must be present at 10^6^ cfu per g. Nonetheless, in Europe, to make a claim for yogurt containing live cultures for improving lactose digestion, the European Food Safety Agency requires a minimum of 10^8^ cfu per g of live bacteria (EFSA Panel on Dietetic Products, Nutrition and Allergies, 2010). In contrast, in Australia and New Zealand, a minimum of only 10^6^ cfu per g is required (Commonwealth of Australia Gazette, 2015).

For many years, cultured dairy products were the only fermented foods that included label declarations regarding the presence of live microorganisms. Label declarations on sauerkraut or kimchi or miso, had, until recently, been rare. The popularity of artisan-style fermented foods (Johnson, 2016) and interest in their health properties (Marco et al., 2017) has led more manufacturers to inform consumers, via food labels, that their products contain live microorganisms. In some cases, the species in these types of foods have been identified and then compared to label claims (Yeung et al., 2002; Scourboutakos et al., 2017). However, to our knowledge, data on the actual levels of live microorganisms in most fermented retail products has not readily been reported or summarized in an organized form. Therefore, consumers, despite their interest in probiotics and functional fermented foods (Linares et al., 2017), have had little access to this useful information.

## Survey design

The purpose of this study, therefore, was to survey the scientific literature and identify published papers in which the number of live microorganisms in a range of fermented foods was reported. Included were so-called western-fermented foods such as yogurt, cheese, and sausage, as well as soy-based and cereal-based fermented foods that are widely consumed in other regions (Tamang et al., 2016). We then organized and summarized the quantitative data from those reports. Our interest was focused on those reports in which foods were obtained from retail locations or were made under manufacturing conditions. Thus, reports describing results from experimentally-produced fermented foods on a laboratory or pilot scale were excluded, in part because they do not reflect commercial processing, distribution, and storage conditions as do retail products. A large number of the reports in the literature in which levels of microbes in fermented foods were described were of this sort. In addition, many reports have analyzed the importance of microbial food safety and hygienic conditions of fermented food products and have reported the presence of spoilage microorganisms or food pathogens. However, the organisms responsible for fermentation and that are commonly present in the finished products were the focus of this current study.

### Search criteria

Scientific articles were chosen that satisfied specific parameters relevant to our stated goals. Specifically, our database search (Google Scholar, WorldCat, Scopus, and PubMed) focused on those studies that enumerated microorganisms exclusively in fermented food products. Keywords for these searches included, but were not limited to, the type of fermented food analyzed and, “commercially produced,” “commercial product,” “enumerated,” “lactic acid bacteria,” “microbial characterization,” “probiotic,” and “culture.” Food products that served only as vehicles for delivery of probiotic microorganisms were not included. Thus, studies that reported counts for frozen yogurt were included, but studies on ice cream containing probiotic microorganisms were not. In general, results were only included for commercial products, bought at retail locations, or those experimentally-produced under industrial manufacturing conditions. Thus, strictly experimental products (e.g., made in a laboratory or under small experimental-scale conditions) were not considered. The only exceptions were for products for which little or no data from retail or industrially manufactured sources was available. In those cases, lab- or pilot-scale-produced products were included, provided they were made using traditional manufacturing methods. No restrictions for date, location, or language were applied.

### Data reporting

For most products, quantitative data relied on cultural methods using well-established types of differential, selective, and general purpose media, as well as appropriate incubation conditions. LAB were the main group described, although other bacterial groups were occasionally reported. Some studies reported single microbial counts, whereas other reported ranges. Although papers reported counts either as log or as actual values, all of the data described in this review are shown as logs. For some products, values were estimated from graphs or figures. When products were held for shelf-life or aging studies, the counts from multiple times points are shown. Otherwise, single time-point data was reported. The region or origin of product manufacture was also noted.

## General survey results

Approximately 400 published studies were reviewed in which fermented foods were characterized for the presence of live microorganisms. However, about three-fourths were excluded and not used in our results. Several excluded studies focused on development of selective methods for distinguishing between different species of LAB, determining ratios (e.g., cocci-to-rods in yogurt), or for enumerating only probiotics organisms. Although most studies reported data based on traditional plating methods, many of the more recent studies reported abundance data (i.e., 16S rRNA-based community sequencing). Because the latter 16S-based methods also detect non-viable cells, these studies were excluded unless total counts were also reported. Ultimately, more than 140 studies were included in our survey. Although the literature from which the results were assembled covers a 50 year period and a range of different regions and methodologies, the results are remarkably consistent. As summarized below, nine groups of fermented foods were reviewed in this survey. These included yogurt and other cultured dairy products, cheese, fermented meats, fermented vegetables, traditional fermented Asian products, fermented cereals, beer, and fermented tea (Kombucha).

### Yogurt and other cultured dairy products

Studies were conducted for retail or commercially manufactured yogurts and other cultured dairy products obtained in the U.S., Australia, Spain, France, Norway, Greece, Argentina, and South Africa (Table 1). All of the yogurts examined contained the yogurt culture organisms, *S. thermophilus* and *L. delbrueckii* subsp. *bulgaricus*, at levels ranging from < 10^4^ to 10^9^ cfu/g or ml. In general, counts for *S. thermophilus* were somewhat higher than for *L. delbrueckii* subsp. *bulgaricus*. In several studies, other microorganisms, including *Bifidobacterium* spp. and *Lactobacillus* spp., were also enumerated. Levels of the latter ranged from undetectable (< 10 cfu/g) to 10^8^ cfu/g. The addition of these probiotic bacteria did not appear to have any effect on levels of the yogurt culture organisms. Although most studies reported counts at only a single time point, other studies reported initial counts as well as at a second time point, usually considered end-of-shelf-life. In such cases, counts were generally similar at both time points (>10^6^ cfu/g), provided samples were stored at refrigeration temperatures (Hamann and Marth, 1984).

**Table 1:** Organisms in commercial yogurt products by region.

**Region**	**Type**	**Source**	**Analyzed microorganisms**	**Initial Count (log cfu/mL or g)**	**Final Count (log cfu/mL or g)**	**Age**	CP^*^	**References**
Argentina	Full and reduced fat yogurt	Retail	*S. thermophilus*	8.87–9.46	–	Within shelf life	6	Vinderola and Reinheimer, 2000
			*L. bulgaricus*^a^	5.58–7.95	–			
			Bifidobacteria^a^	2.60–8.71	–			
			*L. acidophilus*^a^	4.62–8.39	–			
			*L. casei*^b^	8.02–8.33	–			
	Set, skimmed set, drinking, and set with “dulce de leche” yogurt^c^	Industrially manufactured	Total LAB	7.54–8.62	–	Within shelf life	25	Birollo et al., 2000
			*S. thermophilus*	7.72–8.58	–			
			*L. bulgaricus*	7.29–7.38	–			
Australia	Full and reduced fat yogurt^d^	Commercially Manufactured	Streptococci	9.15–9.6	8.79–9.15	After manufacture and by expiration	4	Micanel et al., 1997
			*L. bulgaricus*	9.08	8.36			
			*L. acidophilus*	6.66–8.08	6.38–8.04			
			Bifidobacteria	5.81	7.54			
	Skim milk and regular yogurt^e^	Did not specify	*L. casei*	–	3.41–7.49	Did not specify	2	Ravula and Shah, 1998
	Variety of flavored, natural, and skinny yogurt^f^	Retail	*S. thermophilus*	8.62–9.17	–	After purchase	5	Tharmaraj and Shah, 2003
			*L. bulgaricus*	4.92–7.68	–			
			*L. rhamnosus*	7.36–7.72	–			
			*L. casei*	4.01–5.53	–			
			*B. lactis*	6.36–7.4	–			
			*L. acidophilus*	5.23–7.83	–			
	Variety of flavored yogurts^g^	Retail	*L. acidophilus*	< 2–8.34	< 2–8	After purchase (around 20–30 days before expiration) and at expiration	26 CP from 14 companies	Shah et al., 2000
			Bifidobacteria	< 2–6.86	< 2–6.18			
			*L. casei*	5.65–8.18	< 2–8.08			
	Yogurt^h^	Did not specify	*L. acidophilus*	–	6.56	–	18	Talwalkar and Kailasapathy, 2004
			Bifidobacteria	–	6.54			
			*L. casei*	–	6.38			
	Yogurt^i^^j^	Obtained from manufacturer	*L. acidophilus*	4–8.5	NVO−7.7	After manufacture and 30 days	5	Shah et al., 1995
			*B. bifidum*	3.3–7	NVO−2.5			
China	Yogurt	Retail	S. *thermophilus + Lactobacillus*	–	4.0–8.18	End of shelf life	31	Dong et al., 2014
England	Yogurt^k^	Retail	Bifidobacteria	–	4.9–7.62	Does not specify	8	Iwana et al., 1993
Greece^i^	Greek type yogurt	Obtained from manufacturer	*S. thermophilus*	9.1	8.5	50 days (product shelf life)	1	Alexopoulos et al., 2017
			*L. delbrueckii subsp. bulgaricus*	8.8	7.9			
Italy	Plain stirred style yogurt	Retail	*S. thermophilus*	7.71–8.9	–	10 days after manufacture	11	De Noni et al., 2004
			*L. bulgaricus*	5.48–8.41	–			
	Sweetened stirred style yogurt	Retail	*S. thermophilus*	8.3–9.59	–	10 days after manufacture	11	De Noni et al., 2004
			*L. bulgaricus*	< 4–8.18	–			
South Africa	Low fat, fruit flavored^i^	Obtained from manufacturer	*S. thermophilus*	8.7–9.5	7.9−9.5	Directly after production, and at expiration date	3	Lourens-Hattingh and Viljoen, 2002
			*L. bulgaricus*	7–8.6	5.5–7			
			*L. acidophilus*	7–8.7	4.9–7			
			*B. bifidum*	2–5.2	2.2–4.9			
United States	Custard style yogurt—plain and flavored^i^	Retail	Total LAB	9.1	–	15 days after manufacture	2 CP from 1 manufacturer	Hamann and Marth, 1984
			*S. thermophilus*	9.1	–			
			*L. bulgaricus*	8.1	–			
	Dannon, Breyers, Yoplait, YoBaby, Wal-Mart, and Kroger varieties	Retail	*Lactobacillus*	–	7.68–8.98	before expiration	10	Dunlap et al., 2009
	Flavored yogurt	Retail	*L. bulgaricus*	5.2–8.87	6.15–8.69	0 and 4 weeks after purchase	58 CP/7 brands	Ibrahim and Carr, 2006
			*S. thermophilus*	7.51–8.94	7.9–8.99			
			Bifidobacteria	< 1–4.7	NVO^**^			
	Plain nonfat yogurt	Retail	*S. thermophilus*	8.14–9.83	–	After manufacture	3	Laye et al., 1993
			*L. bulgaricus*	9.04–9.33	–			
	Stirred style yogurt–flavored^i^	Retail	Total LAB	8.6	–	6 days after manufacture	1	Hamann and Marth, 1984
			*S. thermophilus*	8.6	–			
			*L. bulgaricus*	7.3	–			
	Yogurt	Retail	Total LAB	-	7.2–8.1	At expiration date	2	Shin et al., 2000
			Bifidobacteria	–	6.5–7.1			

* *CP, commercial products*.

** NVO, No viable organisms.

a Only viable in 4 of 6 CP.

b Only viable in 3 of 6 CP.

c Reported as average on duplicate agar plates.

d L. delbrueckii spp. bulgaricus—reported in only one product. L. acidophilus −1 of 4 CP had NVO. Bifidobacteria−1 of 4 CP had NVO and 1 product had no detectable counts at initial enumeration (week 0).1

e Lower end of range are microbial counts for skim milk yogurt and higher end are for regular yogurt. Both products claimed to contain L. casei.

f S. thermophilus —Seen in 5 of 5 CP, “yogurt culture” claimed in all 5 CP. L. bulgaricus—Seen in 2 of 5 CP, “yogurt culture” claimed in all 5 CP. L. rhamnous—Claimed in 2 of 5 CP. L. casei—Claimed in 2 of 5 CP. B. lactis—Claimed in 4 of 5 CP. L. acidophilus—Claimed in 4 of 5 CP.

g L. acidophilus—Claimed in 24 CP. Bifidobacteria—Claimed in 18 CP. L. casei—Claimed in 8 CP.

h L. acidophilus—9 of 18 CP. Bifidobacteria-−8 of 18 CP. L. casei—6 of 18 CP.

i Interpreted from graph.

j L. acidophilus—2 of 5 CP had NVO. B. bifidum—4 of 5 CP had NVO.

k *Observed in 5 of 8 CP, claimed in all products*.

In addition to fresh yogurt, frozen yogurt was also examined for bacteria. Results from several studies indicates that when these products were assessed for the relevant yogurt LAB, levels were generally similar to fresh yogurt, with counts ranging from 10^4^ to 10^9^ cfu/g. The stability of lactic cultures in frozen yogurt during long-term storage at freezer temperature (-23 C) has also been studied (Lopez et al., 1998). In general, LAB (*S. thermophilus* and *L. delbrueckii* subsp. *bulgaricus*) survived beyond the designated shelf-life period (1 year), with less than a 0.5 log reduction for most samples.

The number and type of live microorganisms in other cultured dairy products have also been reported (Table 2). These include kefir, cultured buttermilk and simply “fermented milk.” As for other cultured dairy products, populations of LAB were in the 10^5^–10^9^ cfu/g range.

**Table 2:** Organisms in commercial cultured dairy products separated by product.

**Dairy product**	**Region**	**Source**	**Analyzed microorganisms**	**Initial Count (log cfu/mL or g)**	**Final Count (log cfu/mL or g)**	**Age**	CP^*^	**References**
Amasi	South Africa	Retail	LAB	5.1–6.29	–	Did not specify	5	Moyane and Jideani, 2013
			Total bacteria count	3.62–4.96	–			
Cultured Buttermilk	Ethiopia^a^	Dairy farms and processing units	Lactococci	6.07–9.25	–	Does not specify	16	Gebreselassie et al., 2016
			Lactobacilli	6.07–8.61	–			
	India	Restaurant	Total viable count	6	–	Does not specify	1	Jayashree et al., 2013
	United States	Retail	Total bacteria count	7.3–8.64	6.08–7.24	After purchase and 7 days after	8	Vasavada and White, 1979
Fermented Milk	Argentina	Retail	*S. thermophilus*	9.11–9.49	–	Within shelf life	2	Vinderola and Reinheimer, 2000
			*L. acidophilus*	4.62–6.60	–			
	Spain	Commercially Manufactured	*S. thermophilus*	8.42	8.37	After manufacture and at shelf life (24 days)	50	Medina and Jordano, 1994
			*L. bulgaricus*	7.71	6.87			
			Bifidobacteria	6.87	6.62			
	Spain^b^	Retail	*S. thermophilus*	9	7	30 days	10	Gueimonde et al., 2004
			*Lactobacillus*	7–7.3	5.1–6.8			
			Bifidobacteria	5.6–7.5	4.1–7.6			
	Spain	Retail	*S. thermophilus*	9.27	–	Within shelf life (28 days)	1	García-Cayuela et al., 2009
			*L. bulgaricus*	7.64	–			
			*L. acidophilus*	6.65	–			
			*L. casei*	6.79	–			
			*B. lactis*	8.2	–			
Frozen Yogurt	France	Obtained from manufacturer^c^	*S. thermophilus*	8.19	–	5 weeks after manufacture	1	Lopez et al., 1998
	Spain	Obtained from manufacturer	*S. thermophilus*	7.57–7.58	–	1 week after manufacture	2	Lopez et al., 1998
			*L. bulgaricus*	4.29–6.79	–			
	United States	Variety of flavors soft/hard from retail and the manufacturer^d^	Total bacteria	< 5.52–8.81	–	Does not specify	34	Kosikowski, 1981
		Vanilla flavors from retail^e^	LAB	6.11–9.32	–	Does not specify	10	Schmidt et al., 1997
		Variety of flavors from retail	Total viable bacteria	2.30–8.53	–	Within shelf life	19	Tieszen and Baer, 1989
Kefir	Greece^f^	Retail	Yeast	5	–	15 days before expiration	9	Kalamaki and Angelidis, 2017
	Korea	Manufactured with commercial grain	LAB	9.62	–	After fermentation	–^g^	Kim et al., 2015
			*Acetic acid bacteria*	9.52	–			
			*Yeast*	7.67	–			
	Norway ^b^^h^	Obtained from TINE Meieret dairy company	*Leuconostoc*	7.1	6.3	After production and at expiration	5	Grønnevik et al., 2011
			*Lactobacillus*	8.1	6.4			
			*Lactococcus*	8.1	5.8			
			Yeast	3.3	3.9			
	Turkey	Retail	*Lactobacillus*	6.51–8.01	–	Does not specify	4	Kesmen and Kacmaz, 2011
			*Lactococcus*	7.53–8.30	–			
	United States^i^	Manufactured with commercial starter culture	*Lactobacillus*	9.15	–	After fermentation	–^g^	OBrien et al., 2016
			*Lactococcus*	9	–			
			Yeast	7.2	–			

* *CP, Commercial Products*.

a Analyzed sour cream buttermilk and sour milk buttermilk.

b *Interpreted from graph*.

c *No significant decrease in S. thermophilus over time. L. bulgaricus was absent in this CP*.

d *Only 23 CP of 34 CP had viable organisms*.

e *NVO in 6 CPs (< 1 log)*.

f *Only viable counts seen in 8 of the 9 CPs*.

g Lab-scale fermentation with commercial kefir grain/starter

h *Presumptive (95:5 ratio) for lactobacillus and lactococcus*.

i *Reported as average from triplicate agar plates*.

### Cheese

Although considerable microbiological data for cheese exists, most of these reports are concerned with microorganisms having public health or cheese quality implications. Still, levels of lactic acid and related bacteria were reported for more than 30 types of cheese from 18 countries including the United States, Italy, France, Germany, Mexico, Ireland, and South Africa (Table 3). Many papers reported the microorganisms as mesophilic streptococci, lactococci, and lactobacilli or as thermophilic streptococci and lactobacilli. Others reported total microorganisms and total LAB. For most products, only one time period was recorded (usually the most aged sample). Microbial counts ranged from undetectable (< 10^3^ cfu/g) to 10^9^ cfu/g, with the highest levels found in Tilsit cheese (typically aged 2–4 months). In contrast, Grana Padano aged 1 year, Parmesan aged greater than 1 year, and Swiss Gruyere aged greater than 1 year all showed no detectable microorganisms (< 10^3^ cfu/g). As noted for other products, the methods used by the investigators may have influenced the reported data. Thus, enumeration of selected organisms (e.g., *S. thermophilus*) was only possible if the appropriate medium and growth conditions were used.

**Table 3:** Organisms in commercial cheese separated by product.

**Cheese**	**Region (Type)**	**Source**	**Analyzed microorganisms**	**Count (log CFU/g)**	**Age**	CP^*^	**References**
Afuega'l Pitu	Spain	Traditionally manufactured	Total viable bacteria count	8.06	60 days	2	Cuesta et al., 1996
			Lactococci	6.77			
			Leuconostocs	6.76			
			Lactobacilli	8.01			
Armada^*^	Spain	Traditionally manufactured	Aerobic Mesophiles	4.39–8.14	16 weeks	2	Tornadijo et al., 1995
			Lactococci	4.17–6.38			
			Lactobacilli	4.19–8.09			
			Leuconostocs	3.38–7.58			
Asiago	Italy (Asiago Allevo)	Commercial sample	Meso. streptococci	5.7	3–10 months	1	Gatti et al., 1999
			Therm. streptococci	8.9			
			Meso. lactobacilli	4.5			
			Therm. lactobacilli	7.2			
*Blue Cheese^b^*	United States	Retail	Total plate count	7.32	Within shelf life	1	Genigeorgis et al., 1991
Brie	Italy	Commercial samples	Meso. streptococci	5.3	1–2 months	1	Gatti et al., 1999
			Therm. streptococci	<3			
			Meso. lactobacilli	n.d.^**^			
			Therm. lactobacilli	<3			
	South Africa^a^	Commercially manufactured	LAB	7–8.8	8 weeks	8	Viljoen et al., 2003
Burgos	Spain	Retail	LAB	4.6–8.8	Time of purchase	36	Garcia et al., 1987
Cabrales	Spain	Obtained from manufacturers	Aerobic mesophiles	7.45–8.36	90 days	2	Flórez et al., 2006
			Lactococci	7.44–8.12			
			Lactobacilli	5.85–7.15			
			Leuconostoc spp.	5.40–6.14			
		Obtained from manufacturers^c, d^	Total viable count	6.8–7.9	120 days	2	Nuñez, 1978
			Streptococci	3.5–5.9			
			Leuconostocs	3–3.8			
			Lactobacilli	3.2–6.5			
			Yeast+Molds	4.1–7.2			
Camembert^a^	South Africa	Commercially manufactured	LAB	7.6–8.5	8 weeks	8	Viljoen et al., 2003
Cheddar	Ireland	Commercially manufactured	*L. paracasei*	8	39 weeks	3	Fitzsimons et al., 2001
	Ireland^c^	Obtained from manufacturer	NSLAB^***^	1.70–6.90	8 weeks	8	Jordan and Cogan, 1993
			NSLAB	6.15	52 weeks	2	
	U.S.^e^	Traditionally manufactured with commercial starter culture	*Lactobacillus*	5.1	180 days	–	Madkor et al., 2000
Colby^b^	United States	Retail	Total plate count	7.6	Within shelf life	1	Genigeorgis et al., 1991
							
Comte	France^f^	Obtained from manufacturer	*Lb. paracasei*	6.28–7.59	168–280 days	4	Depouilly et al., 2004
			*Lb. rhamnosus*	5.37–6.9			
	Switzerland^c^ ^g^	Commercially manufactured	Thermophilic streptococci	6.75	24 weeks	3	Bouton et al., 1998
			Thermophilic lactobacilli	7			
			Facultative heterofermentative lactobacilli	7.5			
			Propionibacteria	7.75			
Danbo	Denmark	Industrially manufactured	*Lactococcus*	5.76	6 weeks	1	Gori et al., 2013
			*Lactobacillus*	5.82–5.87			
Edam	Egypt (Edam-like cheese)^h^	Manufactured with commercial starter culture	Total viable bacteria count	7.76	15 weeks	1	Ayana and El-Deeb, 2016
	Italy	Commercial samples	Meso. streptococci	2.9	1–2 months	1	Gatti et al., 1999
			Therm. streptococci	4.3			
			Meso. lactobacilli	5.8			
			Therm. lactobacilli	5.3			
Feta	Greece	Obtained from manufacturer^b^	LAB	6.1	60 days	1	Alexopoulos et al., 2017
		Retail^i^	*Lactobacillus*	5.95–7.19	>60 days	4	Rantsiou et al., 2008
			*Lactococcus*	4.18– < 5			
	Iran (Probiotic feta)	Commercially manufactured	*Lactobacillus acidophilus*	6.7	Did not specify	1	Mohammadmoradi et al., 2015
			*Bifidobacterium lactis*	6.7			
Fontina	Italy	Commercial sample	Meso. streptococci	8.3	3–10 months	1	Gatti et al., 1999
			Therm. streptococci	8.3			
			Meso. lactobacilli	4.6			
			Therm. lactobacilli	8.6			
	Italy (Fontal)	Commercial samples	Meso. streptococci	<3	1–2 months	1	Gatti et al., 1999
			Therm. streptococci	5.2			
			Meso. lactobacilli	<3			
			Therm. lactobacilli	4.4			
Galotyri^h^	Greece	Retail	Total viable count	8.03	Time of purchase	1	Samelis and Kakouri, 2007
			Lactobacilli	7.55			
			Lactococci	8.11			
Gorgonzola	Italy	Commercial sample	Meso. streptococci	3.5	3–10 months	1	Gatti et al., 1999
			Therm. streptococci	7.4			
			Meso. lactobacilli	3.1			
			Therm. lactobacilli	6.4			
		Obtained from manufacturer^d^	Total mesophilic bacteria	7.36–7.56	86 days	1	Gobbetti et al., 1997
			*S. thermophilus*	7.85–7.92			
			*Lb. delbrueckii* subsp. *bulgaricus*	3.67–5.77			
			Mesophilic lactobacilli	5.57–5.69			
			Lactococci	7.73–7.87			
			Mold	6.81–7.44			
Gouda	Belgium^k^	Commercially manufactured	Total microflora count	5.8	42 days	1	Messens et al., 1999
			LAB	7.1			
			*Lactococcus lactis*	6.1			
	Belgium (Bellie)^c^	Commercial starter culture	*Enterococcus*	6.45–6.90	12 weeks	1	Van Hoorde et al., 2008
			*Lactobacillus*	6.3–7.3			
			*Lactococcus*	7.2–7.7			
			*Leuconostoc*	7.4–7.6			
	Belgium (Dulses)^c^	Commercial starter culture	*Enterococcus*	6.40–6.55	12 weeks	1	Van Hoorde et al., 2008
			*Lactobacillus*	6.90–7.20			
			*Lactococcus*	7.50–7.70			
			*Leuconostoc*	7.60–7.90			
	South Africa	Commercially manufactured	*Lactobacillus*	8.96	32 days	1	Welthagen and Viljoen, 1998
			*Lactococcus*	9.1			
			Total plate count	9.04			
Gubbeen^l^	Germany^m^	Traditionally manufactured with commercial starter culture	Total bacterial count	7.3	16 days	1	Mounier et al., 2006
							
Grana Padano	Italy^n^	Commercially manufactured	*Lactobacillus*	4.94–6.22	9 months	1	Monfredini et al., 2012
			*Lactococcus*	3.15–6.05			
	Italy	Commercial samples	Meso. streptococci	<3	>1 year	3	Gatti et al., 1999
			Therm. streptococci	<3			
			Meso. lactobacilli	<3			
			Therm. lactobacilli	<3			
	Italy	Commercial samples	Meso. streptococci	<3	3 days ripened	1	Gatti et al., 1999
			Therm. streptococci	<3			
			Meso. lactobacilli	4.4			
			Therm. lactobacilli	7			
	Italy^f^	Obtained from manufacturer	*Lactobacillus*	4.53	13 months	6	Santarelli et al., 2013
			Total viable count	7.11			
Havarti	Denmark (Pasteurized milk havarti)	Traditionally manufactured	*Lactococcus*	5.69	12 weeks	1	Gori et al., 2013
			*Lactobacillus*	3.65–5.54			
	Denmark (Raw milk Havarti)	Traditionally manufactured	*Lactococcus*	7.56	12 weeks	1	Gori et al., 2013
			*Lactobacillus*	6.45–7.75			
Livarot	France	Retail	Total bacteria count	8.58	Does not specify	1	Mounier et al., 2009
			Yeast	6.38			
Limburger^b^	United States	Retail	Total plate count	7.98	Within shelf life	1	Genigeorgis et al., 1991
							
Manchego	Spain	Retail	LAB	4.6–10.03	Time of purchase	36	Garcia et al., 1987
		Manufactured with commercial starter culture^c^	*Lactococcus*	5.9	150 days	1	Poveda et al., 2003
			*Lactobacillus*	5.5			
Monterey Jack^b^	United States	Retail	Total plate count	>6.0–8.62	Within shelf life	4	Genigeorgis et al., 1991
Mozzarella	Italy	Commercial Samples	Meso. streptococci	6.3	< 20 days	1	Gatti et al., 1999
			Therm. streptococci	7.6			
			Meso. lactobacilli	4.3			
			Therm. lactobacilli	<3			
	Italy (Buffalo milk)	Retail	LAB	4.82	Within expiration date	18	Pisano et al., 2016
	Italy (Mozzarella Bufala)	Commercial samples	Meso. streptococci	5.6	< 20 days	1	Gatti et al., 1999
			Therm. streptococci	5.6			
			Meso. lactobacilli	4.8			
	Italy (Mozzarella Bufala Campana)	Local markets	LAB	4.0–7.8	Within shelf life	3	Devirgiliis et al., 2008
	Italy (Cow milk)	Commercially manufactured with commercial starter	Therm. lactobacilli	4.6	15 days	1	De Angelis et al., 2008
			Meso. lactobacilli	4.81			
			*Streptococcus*	7.85			
			*Enterococcus*	3.87			
	Italy (Cow milk)	Retail	LAB	7.08	Within expiration date	14	Pisano et al., 2016
Muenster^b^	United States	Retail	Total plate count	4.53	Within shelf life	1	Genigeorgis et al., 1991
Parmesan	Italy (Parmigiano Reggiano)	Obtained from manufacturer	LAB	7.52	150 days	15	Coppola R. et al., 2000
	Italy (Parmigiano Reggiano)	Commercially manufactured	LAB	6.18	2 months	1	Gatti et al., 2008
			LAB	2.3	24 months		
	Italy (Parmigiano Reggiano)	Commercial samples	Meso. streptococci	<3	>1 year	1	Gatti et al., 1999
			Therm. streptococci	<3			
			Meso. lactobacilli	<3			
			Therm. lactobacilli	<3			
Puzzone di Moena^o^	Italy	Traditionally manufactured	*Lactobacillus*	7.1–7.7	3 months	2	Franciosi et al., 2008
			*Lactococcus*	7.5–7.7			
Pecorino Romano	Italy	Commercial sample	Meso. streptococci	3.5	3–10 months	1	Gatti et al., 1999
			Therm. streptococci	5.5			
			Meso. lactobacilli	3.7			
			Therm. lactobacilli	3			
Provolone	Italy (Piquant provolone)	Commercial sample	Meso. streptococci	2.5–3.4	3–10 months	2	Gatti et al., 1999
			Therm. streptococci	5.4–8.3			
			Meso. lactobacilli	2.8– < 3			
			Therm. lactobacilli	5.5–7.2			
	Italy (Sweet provolone)	Commercial sample	Meso. streptococci	< 3–4.3	3–10 months	2	Gatti et al., 1999
			Therm. streptococci	4.5–7.1			
			Meso. lactobacilli	<3			
			Therm. lactobacilli	< 3–7.1			
Queso Fresco^p^	Mexico	Obtained from manufacturer	Mesophilic streptococci	6.85–9.07	Within 5 days of manufacturer	6	Renye et al., 2008
			Thermophilic streptococci	5.04–9.02			
			Mesophilic lactobacilli	7.13–8.99			
			Thermophilic lactobacilli	5.01–9.01			
			*Leuconostoc*	5.86–9.23			
			*Enterococcus*	5.05–7.91			
Serrano^l^	Brazil	Retail	*Lactococcus*	8.60–9.10	Within shelf life	10	Delamare et al., 2012
			*Lactobacillus*	7.95–9.10			
Stilton	United Kingdom^q^	Retail	Mesophilic LAB	8.87	Within shelf life	16	Ercolini et al., 2003
			*Lactobacillus*	7.76			
			Mesophilic streptococci	8.97			
			Mesophilic, anaerobic LAB	8.85			
	United Kingdom (blue-veined raw milk cheese)^d^	Obtained from manufacturer	LAB	6.90–7.41	After aging (12 weeks)	1	Yunita and Dodd, 2018
			*Lactobacillus*	4.85–6.18			
			*Lactococcus*	7.83–8.65			
Swiss^c, r^	France	Traditionally manufactured	Propionibacteria	7.5–7.6	24 weeks	2	Demarigny et al., 1996
			Facultatively heterofermentative *Lactobacillus*	7.4–7.9			
			Thermophilic streptococci	3.0–5.6			
			Thermophilic lactobacilli	2.6–5.9			
Swiss Gruyere	Italy	Commercial sample	Mesophilic streptococci	<3	>1 year	1	Gatti et al., 1999
			Thermophilic streptococci	<3			
			Mesophilic lactobacilli	<3			
			Thermophilic lactobacilli	<3			
Tilsit	Austria	Obtained from manufacturer	Total bacterial count	8.4–9.7	21 days	13	Eliskases-Lechner and Ginzinger, 1995

* *CP, Commercial Products*.

** *n.d., not determined*.

*** *NSLAB, non-starter LAB count*.

a *Winter and summer cheese analyzed on surface and in center*.

b *Did not support L. monocytogenes surface growth when enumerated*.

c *Interpreted from graph*.

d *Surface and interior of cheese was analyzed*.

e *Lactobacillus count of control cheese (not adjunct culture added)*.

f *Lb. rhamnosus and Lb. paracasei were the only microorganisms enumerated in all 4 CP*.

g *Average of CP*.

h *The control from an Edam-like cheese experiment of goat's diet*.

i *3 of 4 CP reported “not applicable” (< 5 log cfu/g)*.

^j^Industrial Cheese with commercial starter cultures

k *Pressure treatment of 0.1 MPa*.

l *Only licensed cheeses analyzed*.

m *Measurement of bacterial growth on cheese surface*.

n *Grana Trentino cheese; Measurements from middle section and core*.

o *Winter and summer cheese at 30°C*.

p *Raw and pasteurized milk cheese*.

q *Reported as average of triplicate agar plates*. *^r^Raw and microfiltered milk reported*.

### Fermented meats

Microbial counts for fermented sausages are shown in Table 4. In general, samples were either obtained from retail, directly from manufacturers, or were produced via industrial conditions. Most samples were from the United States, Spain, Portugal, and Italy and were composed of pork and/or beef. The levels of microorganisms (LAB and total) ranged from undetectable (< 10^2^ cfu/g) to 10^10^ cfu/g. Data were reported as either within the product shelf life or after ripening or maturation of the sausage. Counts of viable microorganisms in sausages from the United States were generally lower (< 10^7^ cfu/g) compared to sausages from other countries. In particular, LAB levels were all < 10^6^ cfu/g. In contrast, several of the European sausages contained high levels of LAB (>10^8^ cfu/g.). European sausages were more often artisan sausages from smaller manufacturers, although similar microorganisms are used in comparison to sausages from the United States.

**Table 4:** Organisms in commercial sausage products by region.

**Country**	**Type**	**Source**	**Analyzed microorganisms**	**Count (log CFU/g)**	**Age**	**CP**	**References**
France	Dry fermented sausage	Obtained from manufacturer	LAB	6.50–7.74	End of drying (9 weeks)	1	Chevallier et al., 2006
Greece	Dry fermented sausage	Obtained from manufacturer	LAB	7.63–8.20	28 days after formulation	1	Samelis et al., 1994
		Commercially produced^a^	LAB	8.1–8.2	End of curing period	2	Papamanoli et al., 2003
Italy	Ciauscolo salami	Commercially produced^a^	LAB	7.5	End of ripening (45 days)	1	Aquilanti et al., 2007
			Yeast	5.5			
		Obtained from manufacturer	Total bacteria	2.7–5.95	End of ripening	22	Silvestri et al., 2007
			LAB	6.77–8.65			
	Fermented Sausage, Friuli Venezia Giulia region	Commercially produced^a^	Total bacteria	6.1	End of ripening (45 days)	1	Cocolin et al., 2001
			LAB	8.3			
		Commercially produced^b^	Total aerobic count	6.62–9.11	End of ripening (21 days)	3	Comi et al., 2005
			LAB	8.39–8.47			
		Obtained from manufacturer	Total bacteria	4.19–9.11	End of maturation	3	Rantsiou et al., 2005
			LAB	8.34–8.78			
	Salami bergamasco	Obtained from manufacturer	Total bacteria	6–7.17	After maturation of 60 days	2	Cocolin et al., 2009
			LAB	9–9.14			
	Salami Brianza	Local markets	Mesophilic lactobacilli	8.6	After purchase	1	Di Cagno et al., 2008
	Salami cremonese	Obtained from manufacturer	Total bacteria	5.17–6.69	After maturation of 60 days	5	Capita et al., 2006
			LAB	7.54–9.38			
	Salami Mantovano	Obtained from manufacturer	Total bacteria	4.23–9.87	After maturation of 60 days	4	Capita et al., 2006
			LAB	7.6–9.38			
		Commercially produced^c^	Lactobacilli	8.01–8.73	End of ripening (60 days)	2	Pisacane et al., 2015
	Salami Milano	Obtained from manufacturer	LAB	8.0	End of ripening (60 days)	1	Rebecchi et al., 1998
	Salami Napoli	Obtained from manufacturer^a^	Mesophilic lactobacilli	6.7	End of ripening (30 days)	1	Coppola et al., 1995
		Commercially produced^d^	Mesophilic LAB	5.5	End of ripening (41 days)	1	Coppola S. et al., 2000
	Salami Piacentino	Local markets	Mesophilic lactobacilli	8.3	After purchase	1	Di Cagno et al., 2008
		Obtained from manufacturer^e^	LAB	8.02–8.84	End of ripening (63 days)	6	Połka et al., 2015
	Salami Piedmontese	Commercially produced	LAB	7.84	End of ripening (45 days)	1	Greppi et al., 2015
	Salami Varzi	Local markets	Mesophilic lactobacilli	8.6	After purchase	1	Di Cagno et al., 2008
	Salsiccia Basilicata^a^	Commercially produced	LAB	4–7.23	End of ripening (40 days)	10	Parente et al., 2001
			Yeast	6–6.6			
	Soppressata Basilicata^a^	Commercially produced	LAB	8–8.4	End of ripening (40 days)	9	Parente et al., 2001
			Yeast	5.2–7			
	Soppressata Molisana^a^	Commercially produced	LAB	8.4	End of ripening (28 days)	2	Coppola et al., 1998
Spain and Portugal	Alheiras	Retail	LAB	5.9–10.5	Within shelf life	12	Capita et al., 2006; Ferreira et al., 2006
	Androlla	Obtained from manufacturer	Total aerobic mesophilic bacteria	7.81–9.52	After 20–30 days of ripening	20	García Fontán et al., 2007b
			LAB	8.78–9.36			
	Botillo	Obtained from manufacturer	Total aerobic mesophilic bacteria	7.63–9.37	After 15–20 days of ripening	15	García Fontán et al., 2007a
			LAB	8.34–9.56			
	Chorizo Ostrich	Retail	Total bacteria	7.3	Within shelf life	8	Capita et al., 2006
			LAB	6.23			
	Chorizo Deer	Retail	Total bacteria	5.46	Within shelf life	6	
			LAB	5.15			
	Chorizo Pork	Retail	Total bacteria	8.25	Within shelf life	18	
			LAB	8.46			
	Salchicón Ostrich	Retail	Total bacteria	6.09	Within shelf life	22	
			LAB	5.61			
	Salchicón Deer	Retail	Total bacteria	6.28	Within shelf life	8	
			LAB	6.26			
	Salchicón Pork	Retail	Total bacteria	8.09	Within shelf life	19	
			LAB	7.5			
United States	Dry salami	Retail	Total bacteria	3–6	Does not specify	11	Acton and Dick, 1976
			LAB	3–5			
	Genoa salami	Retail	Total bacteria	3–7	Does not specify	8	
			LAB	2–6			
	Lebanon bologna	Retail	Total bacteria	7–8	Does not specify	5	
			LAB	< 3			
	Pepperoni	Retail	Total bacteria	4–7	Does not specify	14	
			LAB	2–6			
	San Francisco dry salami	Retail	Total bacteria	6–7	Does not specify	4	
			LAB	3–6			
	Semidry salami	Retail	Total bacteria	3–4	Does not specify	8	
			LAB	< 2			
	Summer sausage	Retail	Total bacteria	3–4	Does not specify	19	
			LAB	4			
	Thuringer	Retail	Total bacteria	3–7	Does not specify	13	
			LAB	5–6			

a Interpreted from graph.

b *Three seasons were analyzed*.

c Crespone casings and Gentile casings were used.

d Core and edge data reported.

e *With and without commercial starter cultures*.

### Fermented vegetables

Microbial counts for fermented vegetables, including sauerkraut, olives, mustard pickles, pickles, and kimchi are summarized in Table 5. Fermented cucumbers products were also considered (listed as pickles). Laboratory-manufactured products, using industrial or traditional practices, were included due to the lack of literature on fermented vegetables from retail sources.

**Table 5:** Organisms in fermented vegetables separated by product.

**Product**	**Region (Type)**	**Source/Fermentation style**	**Analyzed microorganisms**	**Count (log cfu/mL or g)**	**Age**	**References**
Kimchi	Taiwan^a^	Supermarkets	Aerobic bacteria	1–7.2	Within shelf life	Tsai et al., 2005
	South Korea	Industrially produced with a spontaneous fermentation^b^^,^ ^c^	*Leuconostoc citreum*	7.4	90 days	Cho et al., 2006
			*Leuconostoc gasicomitatum*	8		
			*Weissella koreensis*	8		
			*Lactobacillus sakei*	7.4		
		Retail (online and markets) with starter cultures and spontaneous fermentations	LAB	7.14–9.23	5 days after purchase	Kim et al., 2016
		Obtained from commercial distributors^b^^,^^d^	Total viable bacteria	7.9–8.3	4 weeks of fermentation	Lee et al., 2018
			LAB	7.8–8.3		
		Obtained from commercial distributors^b^^,^ ^e^	Total viable bacteria	7.9	4 weeks of fermentation	Lee M. et al., 2017
			LAB	7.8		
Mustard Pickles	Taiwan^f^	Supermarkets	Aerobic bacteria	< 1.0–4.2	Within shelf life	Kung et al., 2006a
Olives	Greece (Conservolea naturally black olives)	Laboratory manufactured with a spontaneous fermentation	LAB count	7.9	30 days	Panagou et al., 2008
		Laboratory manufactured with a commercial starter culture	LAB count	8	30 days	Panagou et al., 2008
	Italy (Bella Di Cerignola -Debittered green table olives)^b^^,^ ^g^	Commercially manufactured with a spontaneous fermentation	LAB count	5.5	90 days	De Bellis et al., 2010
	Italy (Nocellara del Belice–Spanish-style green olives)^h^	Industrially manufactured with a spontaneous fermentation	Viable cell count	6.58–7.40	131 days	Aponte et al., 2012
			*Lactobacillus*	7.21–7.35		
			Lactic streptococci	6.49–6.95		
	Italy (Nocellara del Belice–green table olives)	Obtained from commercial manufacturer with spontaneous fermentation	LAB	4.53	7–10 months	Romeo et al., 2012
	Portugal (Galega and Cordovil)^b^	Laboratory manufactured with a spontaneous fermentation	Viable LAB count	4.9	150 days	Silva et al., 2011
	Southern Spain (Spanish-style green olives)^b^	Industrially manufactured with a spontaneous fermentation	*Lactobacillus*	5.5	120 days	Ruiz-Barba and Jiménez-Díaz, 2012
			Lactic cocci	NVO^*^	120 days	
		Industrially manufactured with commercial starter culture^b^	*Lactobacillus*	5.9	120 days	Ruiz-Barba and Jiménez-Díaz, 2012
			Lactic cocci	4	120 days	
	United States (Sicilian-style green olive–colossal Sevillano olives)^b^	Commercially manufactured with a spontaneous fermentation	LAB count	7.4	200 days	Golomb et al., 2013
Pickles	India^b^^,^ ^i^	Laboratory manufactured with a spontaneous fermentation	LAB	7.1	3 days	Singh and Ramesh, 2008
	United States^b^^,^ ^j^	Laboratory manufactured with a pure culture fermentation	*P. cerevisiae*	8.26–8.77	Did not specify	Etchells et al., 1964
			*L. plantarum*	8.72–8.96		
			*L. brevis*	7.79–8.45		
Sauerkraut	United States^b^	Commercially manufactured with starter culture	LAB	8.3	10 days	Johanningsmeier et al., 2004
			Heterofermentative LAB	2.7		
	United States^b^	Commercially manufactured with a spontaneous fermentation	Total microbial count	7	60 days	Lu et al., 2003
			LAB	7		
	Croatia^k^	Laboratory manufactured with a spontaneous fermentation	Total microbial count	6.04	42 days	Beganović et al., 2011
			LAB	3.79	42 days	
	Finland^l^	Large-scale manufacturing with a spontaneous fermentation	LAB	7.3	15 days	Viander et al., 2003

* *NVO, No viable organisms*.

a *20 commercial products*.

b *Interpreted from graph*.

c *Incubation of microorganisms were at 15°C*.

d *Three seasons were analyzed*.

e *19 out of 44 Chinese cabbage samples (88 total samples using other vegetables) were provided by commercial suppliers*.

f *14 CP (Commercial Products)*.

g *Data from control set (no inoculation) with 8% NaCl*.

h *Olive from both irrigated and not irrigated fields*.

i *30 cucumber samples were used*.

j *Individual fermentations of each microorganism*.

k *Fermentations with 4% NaCl*.

l *Fermentations with 1.2% NaCl*.

Microbial counts for sauerkraut were generally reported as LAB with counts ranging from 10^3^ to 10^8^ cfu/g. Reported samples were for sauerkraut originating from the United States, Finland, and Croatia. Levels of LAB and *Lactobacillus* were reported for olives produced in Italy, Greece, Portugal, Spain, and the United States. These products contained 10^4^ to 10^8^ cfu/g and were between 30 and 200 days.

Other products for which quantitative data were reported included mustard pickles and kimchi from Taiwan and pickled cucumbers from China, India, and the United States. Microbial counts ranged from undetectable (< 10^1^) to 10^8^ cfu/g. For several of these products, levels of species (e.g., *Lactobacillus plantarum, Lactobacillus brevis*, and *Pediococcus cerevisiae*) were reported. Species of *Leuconostoc, Weissella* and *Lactobacillus* were also reported for Korean kimchi, where they were generally present between 10^7^ and 10^8^ cfu/g.

### Traditional asian fermented products

Another group of fermented foods that contain lactic acid bacteria and other bacteria are those products traditionally manufactured in Asia and that rely on grain or legume substrates. One important difference in the fermentation of these food products compared to other fermented foods is the reliance on fungal enzymes to convert complex carbohydrates to simple sugars. Aerobic conditions are another unique characteristic used in various parts of the fermentation process. Data were collected for several products, including miso, tempeh, fish sauce, and fermented fish (Table 6). Similar to the fermented vegetables, there were few reports on products from retail sources. Therefore, laboratory manufactured products made using industrial or traditional practices were included. In general, aerobic bacteria counts of miso ranged from 10^2^ to 10^7^ cfu/g. Similar bacterial counts were reported for fish sauce. LAB counts for tempeh and fermented fish were between 10^3^ to 10^7^ cfu/g with fermented fish being at the lower end of the range.

**Table 6:** Organisms present in traditional Asian fermented products separated by product.

**Product**	**Region (Type)**	**Source**	**Analyzed microorganism**	**Count (log cfu/g)**	**Age**	**References**
Fermented Fish	Japan (Funazushi—fermented sushi)	Obtained from commercial manufacturer	LAB	3.48–5.43	Does not specify	Tsuda et al., 2012
Fish Sauce	Malaysia (anchovy)^a^	Obtained from commercial manufacturer	Aerobic bacteria	4.92–5.53	6–12 months	Zaman et al., 2010
Miso	Taiwan^b^	Supermarkets	Aerobic bacteria	2.1–7.1	Within shelf life	Kung et al., 2006b
	Japan	Laboratory manufactured with a spontaneous fermentation	Aerobic bacteria	4.3	15 weeks	Onda et al., 2003
Tempeh	Netherlands	Laboratory manufactured with industrial processes and a spontaneous fermentation^c^	LAB	7.01	Does not specify	Nout et al., 1987
		Shops, production places, and restaurants^d^^,^ ^e^	LAB	3–9	24 h after purchase	Samson et al., 1987

a 5 CP.

b *27 CP (Commercial Products)*.

c *Measure of tempeh and not the soak*.

d *81% of samples >10^7^ CFU/g*.

e *110 samples were analyzed*.

### Fermented cereals

Fermented porridges and gruels are widely consumed in many African countries. Here, studies were reported from Burkina Faso, Uganda, Ghana, Benin, Tanzania, and Mexico (Table 7). These cereals were made using pearl millet, millet, sorghum, and maize as starting grains. In general, the cereals contained LAB and mesophilic aerobic bacteria with a range of 10^5^ to 10^9^ cfu/g.

**Table 7:** Organisms in commercial fermented cereals from Africa and Mexico.

**Product (Region)**	**Source**	**Analyzed microorganisms**	**Count (log CFU/g)**	**Grain**	**CP**	**References**
Ben-saalga (Burkina Faso)	Obtained from manufacturer	Total aerobic mesophiles	7.1	Pearl millet	12	Tou et al., 2006
		LAB	7			
		Yeast	5.5			
Bushera (Uganda)	Markets	LAB	8.1–8.4	Millet	5	Muyanja et al., 2003
		LAB	8.4	Sorghum	5	
		LAB	8.9–9	Millet and Sorghum	5	
Fura (Ghana)	Obtained from manufacturer	LAB	6.6–8	Does not specify	8	Owusu-Kwarteng et al., 2012
Koko Sour Water (Ghana)^a^	Obtained from manufacturer	LAB	8	Does not specify	3	Lei and Jakobsen, 2004
Mawè (Benin)	Market and manufacturer	Total aerobic mesophiles	8.8	Does not specify	30	Hounhouigan et al., 1993
		LAB	8.9			
		Yeast	6.4–6.9			
Pozol (Mexico)^b^	Market	Total bacteria	9.5	Does not specify	1	Omar and Ampe, 2000
		LAB	9			
Togwa (Tanzania)^c^	Obtained from manufacturer	LAB	9	Sorghum, maize, millet, and maize	36	Mugula et al., 2003
		Yeast	7			

a *Koko is porridge that have been heat treated. Koko sour water is the edible untreated water byproduct*.

b *Interpreted from graph. Measured outside and inside of sample in triplicate*.

c *Samples were obtained from manufacturer before fermentation*.

### Beer

Several sour beer products from Belgium, such as lambic and gueuze, were included in the survey (Table 8). LAB counts were reported for these products, ranging from 10^2^ to 10^5^ cfu/g. The age of the products reported in the table refers to the longest time the beer was left to age. This maximum aging time was found to range from 40 days to 5 years across the different products.

**Table 8:** Organisms in commercial sour beer products.

**Product**	**Region**	**Source**	**Analyzed microorganisms**	**Count (log CFU/g)**	**Age**	**References**
Gueuze	Belgium	Obtained from a traditional brewery	LAB	5.25–5.31	2 years	Spitaels et al., 2015a
			LAB	3.87–3.88	4 years	
			LAB	3.49–3.96	5 years	
Lambic	Belgium	Obtained from a traditional brewery^a^	LAB	3.08–4.26	24 months	Spitaels et al., 2014
		Obtained from industrial brewery	LAB	4.33–4.38	12 months	Spitaels et al., 2015b
		Obtained from two breweries^b^	LAB	2.3–2.75	40 days	Martens et al., 1991

a *Incubated at 28°C aerobically or 20°C anaerobically on MRS agar*.

b *Interpreted from graph*.

### Fermented tea (kombucha)

Kombucha is a fermented beverage made from sweetened tea to which a specialized culture is added. The latter is comprised of a *s*ymbiotic *c*ulture *o*f *b*acteria and *y*east or SCOBY, normally within a cellulose-type membrane. Bacteria commonly found in kombucha include the acetic acid bacteria belonging to the genera, *Acetobacter, Gluconacetobacter*, and *Gluconobacter*, as well as LAB. Most of the yeasts associated with kombucha are species of *Saccharomyces*, although other yeast genera may also be present (Teoh et al., 2004; Coton et al., 2017). While this product is now widely consumed, and manufacturers promote the presence of live microorganisms on product labels, there are few published data on the levels of microbes present in retail products. One recent study reported both bacterial and yeast counts for two kombucha products that were produced under industrial manufacturing conditions (Coton et al., 2017). In general, acetic acid bacteria levels ranged from 10^6^ to 10^7^ cfu/mL at the end of the fermentation, and similar counts were reported for LAB and total aerobic bacteria. Total yeast counts of about 10^7^ cfu/mL were also reported.

## Discussion

### Food-associated microbes travel and interact in the gut

The human gastrointestinal tract is home to more than 10^12^ microbes. This diverse ecosystem provides protection against pathogens, extracts nutrients from dietary components, and modulates the immune system (Lozupone et al., 2013). The gut microbiota is also very stable, although several factors, including exposure to antibiotics, stress, and disease can disrupt this community, leading to dysbiosis (Sommer et al., 2017). The ability of diet and dietary components to modulate the gastrointestinal microbiota, redress dysbiosis, and enhance human health is now well- established (David et al., 2014; Graf et al., 2015; Sonnenburg and Bäckhed, 2016).

Among the food components known to influence the composition of the microbiota are fermentable fibers and prebiotics that enrich for particular members of the gut microbiota. Another route by which the gastrointestinal microbiota may be modulated is via consumption of probiotics—live microbes consumed at a dose sufficient to provide beneficial effects (Hill et al., 2014). Probiotics, however, are temporary members of the microbiome and rarely persist more than a few days (Tannock, 2003; Derrien and van Hylckama Vlieg, 2015; Zhang et al., 2016).

Perhaps the easiest and most common way to introduce potentially beneficial microbes to the gastrointestinal tract is via consumption of microbe-containing foods, and fermented foods and beverages, in particular. Like many probiotics, many microbes associated with fermented foods may also have the capacity to survive digestion, reach the gastrointestinal tract, and ultimately provide similar health benefits (Marco et al., 2017). When consumed regularly, these fermentation-associated microbes form what some researchers have called the “transient microbiome” (Derrien and van Hylckama Vlieg, 2015).

In general, the microorganisms present in fermented foods and beverages originate via one of two ways. For so-called natural or spontaneous fermented foods, the microorganisms are autochthonous and are naturally present in the raw material or manufacturing environment. To survive fermentation and processing, the LAB, yeasts, and any other microorganisms present in the finished product must manage a range of selective and competitive pressures, including salt, organic acids, ethanol, anaerobiosis, and low pH. Many of the fermented foods reviewed in this survey, including fermented cereals, sauerkraut, kimchi, and other fermented vegetables, and fermented soy-based products are made by natural fermentation. In addition, many wines and even some fermented sausages and beers are made in this manner.

Other fermented foods rely on the addition of a starter cultures. Cultured dairy products, cheese, and fermented sausages are commonly made using starter cultures. When cultures are used, their selection is based on the performance characteristics specific to the product. In addition, the incubation temperature during fermentation and the nutrient content are usually well-suited to the needs of the microorganisms. In many cases, the culture is added at such high inoculum levels, there would be little competition from other organisms. Collectively, most food fermentation microorganisms are well-adapted to the food environment.

In contrast, once the organisms present in fermented foods are consumed, they become foreign or allochthonous to the gastrointestinal tract. In most cases, they lack the physiological and biochemical resources to compete in this ecological niche. If they survive transit, they do not become stable members of this community (Zhang et al., 2016). Nonetheless, the presence of food fermentation-associated microorganisms in the GI tract, even if they are just “passing through,” is now well-documented (Lee et al., 1996; Walter et al., 2001; Dal Bello et al., 2003; David et al., 2014; Derrien and van Hylckama Vlieg, 2015; Zhang et al., 2016; Lisko et al., 2017).

### Evidence of health benefits associated with fermented foods

The evidence for the potential health benefits of fermented foods is based on numerous epidemiological as well as clinical reports (reviewed in Marco and Golomb, 2016; Kok and Hutkins, in press). In general, epidemiological studies have shown that consumption of fermented foods is associated with improvements of health status or reductions in disease risk. For example, yogurt-rich diets were associated with a reduced risk of metabolic syndrome in older Mediterranean adults (Babio et al., 2015). A similar finding was reported in another large cohort study that showed cultured milk consumption reduced the risk of bladder cancer (Larsson et al., 2008). Yogurt consumption has also been associated with reduced weight gain (Mozaffarian et al., 2011). Epidemiological data also suggests that consumption of other fermented foods may be correlated to beneficial health outcomes. Consumption of kimchi and other fermented vegetables, for example, correlated with reduced incidence of asthma and atopic dermatitis in Korean adults (Park and Bae, 2016; Kim et al., 2017). Reduced risks of type 2 diabetes and high blood pressure among Japanese adults was associated with consumption of fermented soybean foods rich in phytoestrogens and bioactive peptides (Kwon et al., 2010; Nozue et al., 2017). In contrast, the large European Prospective Investigation into Cancer and Nutrition cohort study from the Netherlands reported no association between fermented foods consumption and overall mortality (Praagman et al., 2015).

Although many human clinical studies have assessed the effects of probiotic-containing fermented foods on health biomarkers, fewer randomized controlled trials (RCT) have considered fermented foods alone. Nonetheless, several reports provide evidence that fermented foods, such as kimchi, fermented soy products, and yogurt, can improve relevant biomarkers. For example, kimchi consumption improved fasting blood glucose and other metabolic syndrome symptoms in overweight and obese adults (Kim et al., 2011), and similar improvements were observed in healthy adults (Choi et al., 2013). Consumption of a fermented soybean paste also improved plasma triglyceride levels in obese adults (Lee Y. et al., 2017). Perhaps the strongest evidence is for yogurt and improved lactose tolerance, due to *in vivo* expression and release of β-galactosidase by the yogurt culture microbes, *S. thermophilus* and *L. delbrueckii* subsp. *bulgaricus* (Kolars et al., 1984; Martini et al., 1987; Pelletier et al., 2001; Savaiano, 2014). This is the only approved health claim approved by the European Food Safety Authority (EFSA Panel on Dietetic Products, Nutrition and Allergies, 2010).

As noted previously, some fermented foods could impart health benefits even in the absence of live microorganisms in the finished products. For example, in sour dough bread manufacture, LAB may express phytase enzymes that degrade phytates and therefore enhance mineral absorption (Nuobariene et al., 2015). In the manufacture of red wine, ethanol produced early in the fermentation enhances extraction of polyphenolic compounds from the grape skins. Fermented foods may also contain vitamins and other bioactive molecules produced *in situ* from microbial metabolism that are not present in the original food. Recently, Saubade et al. (2017) noted that folic acid deficiency is a global health problem and suggested that fermented foods could be a food-based alternative for delivering folic acid to at-risk populations. Although some LAB are able to produce modest levels of folate (Leblanc et al., 2011), amounts produced in foods may be too low to be reach required levels (Saubade et al., 2017). Thus, selection of over-producing strains, as well as combining strains with non-LAB may be necessary to enhance production of this vitamin in foods.

If present, fermentation-derived microorganisms, despite their transient nature, may yet have the potential to influence gut microbiota diversity, structure, and function (Zhang et al., 2016). Notably, they may also affect health due to their ability to out-compete pathogens for resources, produce short chain fatty acids from available carbohydrates, secrete anti-microbial agents, contribute to immune homeostasis, and produce vitamins, *in situ* (Derrien and van Hylckama Vlieg, 2015).

### The number of fermentation-associated microbes depends on region and product age

In this survey, we reviewed the literature for studies that included quantitative data on microorganisms present in commercial fermented food products. To our knowledge, this is the first time that there has been a compilation of the hundreds of previous studies that enumerated microbes in fermented foods from retail samples or commercial products. In general, most of the products for which data were available contained at least 10^6^ cells/mL or g. However, there was considerable variation depending on product age and region, and several relevant bacterial species or groups were present at less than that amount.

Although regular consumption of yogurt is often included in dietary guidelines (Smug et al., 2014), recommendations for other fermented foods rarely exist (Chilton et al., 2015). Likewise, to our knowledge, there are few guidelines for what constitutes a minimum dose of live microorganisms. The one exception is the yogurt health claim for “improved lactose tolerance” that was approved in 2010 by the European Food Safety Authority (EFSA Panel on Dietetic Products, Nutrition and Allergies, 2010). The claim states that yogurt should contain at least 10^8^ cfu live starter microorganisms per gram- the same count the NYA requires for the “live and active” seal, as noted above.

Even in the absence of a seal or stamp, many commercial yogurt products, as well as kefir, fermented vegetables, and miso, also provide numerical information on their labels. Recently, Derrien and van Hylckama Vlieg (2015) suggested that consumption of 10^10^ cells would be necessary to induce an effect on the microbiota and host health. This could be achieved by consuming 100 g of fermented food containing 10^8^ cells/g.

**Figure 1:**
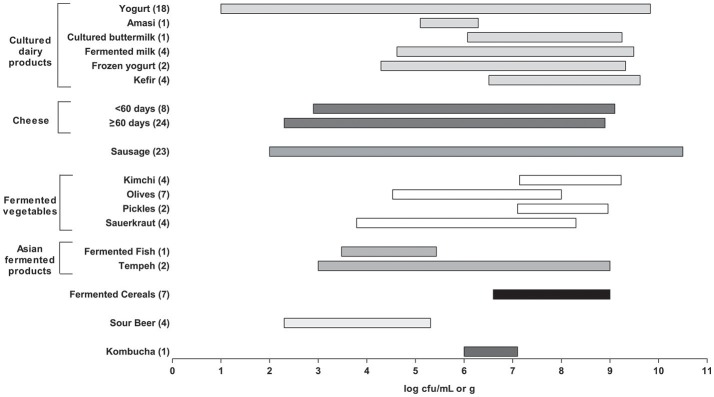
Summary of lactic acid bacteria (LAB) counts in all fermented foods as reported in **Tables 1–8**. The bar plots represents a range (minimum to maximum) of counts found across the studies surveyed. The number of studies used here for each fermented food is shown in brackets. Products were excluded if they had no viable counts or when LAB counts were not reported. For yogurt, initial counts were used for products that had counts for more than one timepoint. For cheese, the products were divided by aging time (60 days) and were excluded if aging time was not reported.

According to the results reported in this survey, many commercial fermented food products would be close to meeting this requirement (Figure 1). However, several caveats are relevant. First, there was a wide range of reported microbial counts (over several logs) within the various product groups. Some products also reported total LAB, whereas other reported specific genera or species or as thermophilic or mesophilic. Second, for most products, enumeration relied on standard cultural methods for LAB (including medium and incubation conditions), which may have under-estimated more fastidious species. This can be attributed to the high stress conditions of fermented products that can occasionally lead to injured microorganisms that are viable but not culturable.

Finally, the age or time at which the products were analyzed also varied considerably. In general, “fresher” products had higher numbers. These would include yogurt and cultured dairy products, as well as kimchi, sauerkraut, and other fermented vegetables. The counts from the cheeses also varied widely, with the longer aged cheeses (e.g., Parmesan, Grana) consistently having the lowest counts.

### Recommendation of fermented foods as part of dietary guidelines

In many cultures, fermented foods containing live microorganisms are consumed on a regular or even daily basis (Hutkins, 2018). Based on the data reported in this survey, consumption of fermented foods would not only provide important macronutrients, they could also deliver large numbers of potentially beneficial microorganisms to the gastrointestinal tract. For example, if Korean kimchi contains 10^8^ lactic acid bacteria per g (Table 5), and given per capita consumption of kimchi is estimated at 100 g per person per day, then the daily consumption of live microbes from kimchi alone would be 10^10^. Likewise, in the Netherlands, where yogurt consumption is also around 100 g per day, similar levels of microbes (i.e., 10^10^ cfu per day) would be ingested. These are the doses noted above that can influence the gut microbiota and provide a potential health benefit (Derrien and van Hylckama Vlieg, 2015).

Recently, the concept of “shared core benefits” was introduced to explain how and why phylogenetically related organisms could deliver similar health benefits (Sanders et al., 2018). Thus, although the microbes in fermented foods cannot, by definition, be considered probiotic, many of them are evolutionarily highly related to probiotic organisms, and they often share the same molecular mechanisms responsible for health-promoting properties in probiotic organisms. The application of various omic approaches to understand functional properties of fermentation-derived microbes will also likely reveal new attributes relevant to the health benefits these microbes may provide (Macori and Cotter, 2018).

In part, this is why several prominent groups have recommended that health care professionals should promote fermented foods containing live microbes as part of public health policy (Ebner et al., 2014; Sanders et al., 2014; Chilton et al., 2015; Bell et al., 2017; Hill et al., 2017). In particular, including fermented foods in dietary guidelines for specific populations has also been recommended. For example, Bell et al. (2018) recently suggested fermented foods should be introduced to children early in life and incorporated into their everyday meal plans. In addition, regular consumption of fermented foods could be especially important for low income, resource-challenged communities that are disproportionally susceptible to gastrointestinal infections (Kort et al., 2015).

## Author contributions

SR, CK, and RH each contributed 30% to data collection. MH contributed 10% to data collection. SR, CK, and RH wrote the manuscript.

### Conflict of interest statement

The authors declare that the research was conducted in the absence of any commercial or financial relationships that could be construed as a potential conflict of interest.
